# Advances in genomics-driven genetic decoding and genomic design breeding in tomato

**DOI:** 10.1093/hr/uhag172

**Published:** 2026-05-04

**Authors:** Zihui Ding, Zhangjun Fei, Sanwen Huang, Yaoyao Wu

**Affiliations:** State Key Laboratory of Crop Genetics & Germplasm Enhancement and Utilization, College of Horticulture, Nanjing Agricultural University, Nanjing 210095, China; Boyce Thompson Institute, Cornell University, Ithaca, NY 14853, USA; National Key Laboratory of Tropical Crop Breeding, Shenzhen Branch, Guangdong Laboratory of Lingnan Modern Agriculture, Genome Analysis Laboratory of the Ministry of Agriculture and Rural Affairs, Agricultural Genomics Institute at Shenzhen, Chinese Academy of Agricultural Sciences, Shenzhen, Guangdong 518120, China; State Key Laboratory of Crop Genetics & Germplasm Enhancement and Utilization, College of Horticulture, Nanjing Agricultural University, Nanjing 210095, China

## Abstract

Tomatoes are highly nutritious and represent one of the important vegetable fruits worldwide. Both historically and moving forward, genetic decoding and precision breeding remain fundamental to tomato improvement. Here, we summarize pivotal advances in decoding tomato genomes across domestication, improvement and evolution processes and provide a perspective on future breeding through precision design. In-depth population genetic studies have revealed how artificial selection systematically prioritized yield-related alleles at the cost of narrowing genetic diversity, especially at flavor-related loci—highlighting the urgent need to reconcile these trade-offs. Comparative genomics across species, viewed through an evolutionary lens, has uncovered critical insights into functional genes, deepening our understanding of the genetic architecture and regulatory mechanisms underlying key traits. Collectively, these advances have enabled precise identification and functional characterization of key genetic elements, paving the way for systematic redomestication of tomato through precision genomic design. Looking ahead, more efficient and precise breeding strategies will be required to accelerate genetic gains in tomato in the coming decades. The integration of recent genomic advances, coupled with genomic selection and artificial intelligence, into genomic design breeding offers a transformative framework, unlocking unprecedented opportunities for developing highly flavorful and consumer-customized tomato varieties.

## Introduction

**Figure 1:**
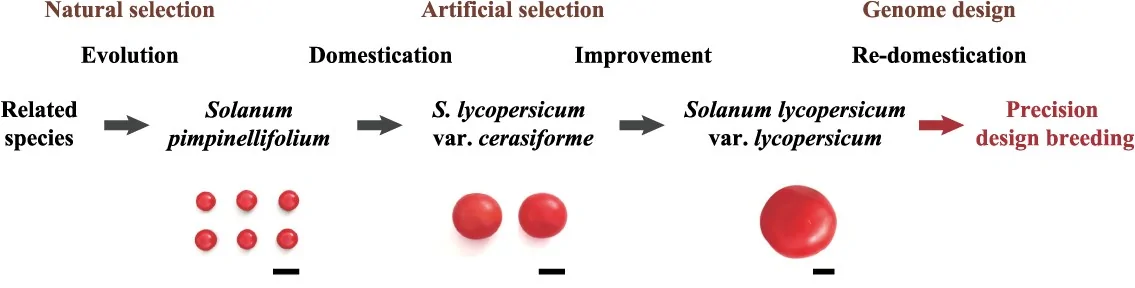
Impact of selection on the tomato genome and its implications for genomic design breeding, illustrating tomato evolution, domestication, and future breeding perspectives. Scale bars, 2 cm.

**Figure 2:**
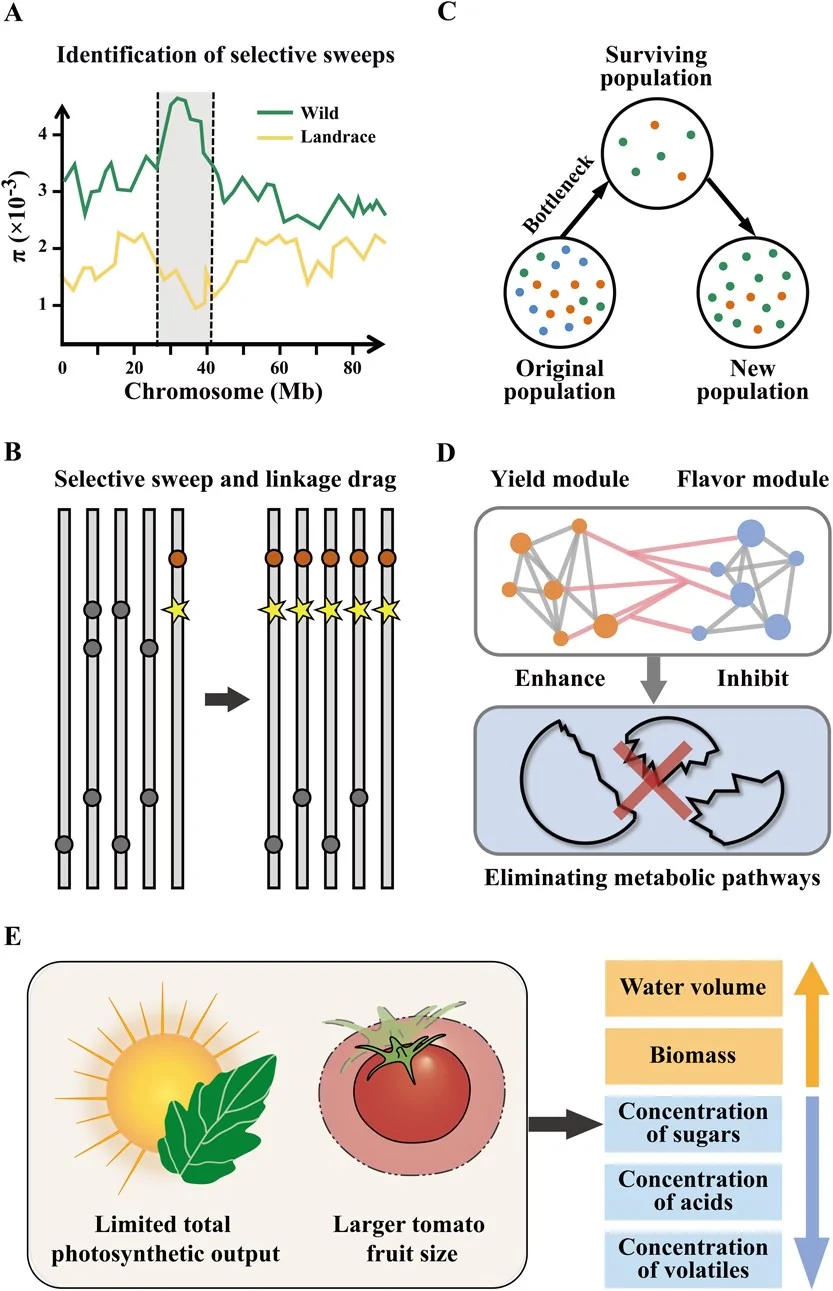
Impact of artificial selection on the tomato genome. (A) Artificial selection leads to the decrease of nucleoid diversity. The region between the two dashed lines indicates a selective sweep region. (B) Selective sweeps lead to the increase in the frequency and sometimes fixation of beneficial alleles (red dots). However, linkage drag between beneficial alleles and deleterious mutations (yellow stars) results in the accumulation of deleterious variants. (C) Population bottleneck reduce overall genetic diversity in the tomato genome. (D) Antagonistic pleiotropy effect between yield and flavor constrains simultaneous improvement of both traits. (E) Limited total photosynthetic products and selection for larger tomato fruits have led to a decrease in the concentration of flavor metabolites.

Tomato (*Solanum lycopersicum*), a member of the Solanaceae family, is one of the world’s most economically important vegetable crops and serves as a key model system for studying fleshy fruit development. Its evolutionary trajectory has been profoundly shaped by both natural and artificial selection, leaving clearly detectable genomic signatures of selective pressure that drove adaptive and fruit-quality changes, ultimately giving rise to modern tomato cultivars (Fig. 1) [1–5]. Over ~10 000 years of human selection, *S. pimpinellifolium* was domesticated into cherry tomato (*S. lycopersicum* var. *cerasiforme*), which subsequently underwent intensive improvement to produce large-fruited modern varieties [5, 6]. This stepwise process of domestication and improvement drove substantial genomic, transcriptomic, and metabolic variation, ultimately shaping the genetic architecture of today’s tomatoes [1, 2, 5, 7, 8]. During domestication and improvement, artificial selection played a pivotal role in shaping the tomato genome by favoring desirable alleles associated with key agronomic traits such as yield and fruit quality (Fig. 1) [1–5, 7]. Such selective pressure led to increased frequencies, or even fixed beneficial alleles at target loci, optimizing tomatoes for human cultivation and consumption while leaving distinct genomic signatures of selective sweeps (Fig. 2A and B). However, these gains were coupled with a severe genetic bottleneck (Fig. 2C) [9–11], which together contributed to the erosion of genetic diversity in modern tomatoes. Moreover, linked selection inadvertently allowed the accumulation of deleterious mutations in the genome, a phenomenon known as the hitchhiking effect (Fig. 2B) [12]. These unintended consequences now present significant challenges for future genetic improvement in tomato breeding.

**Figure 3:**
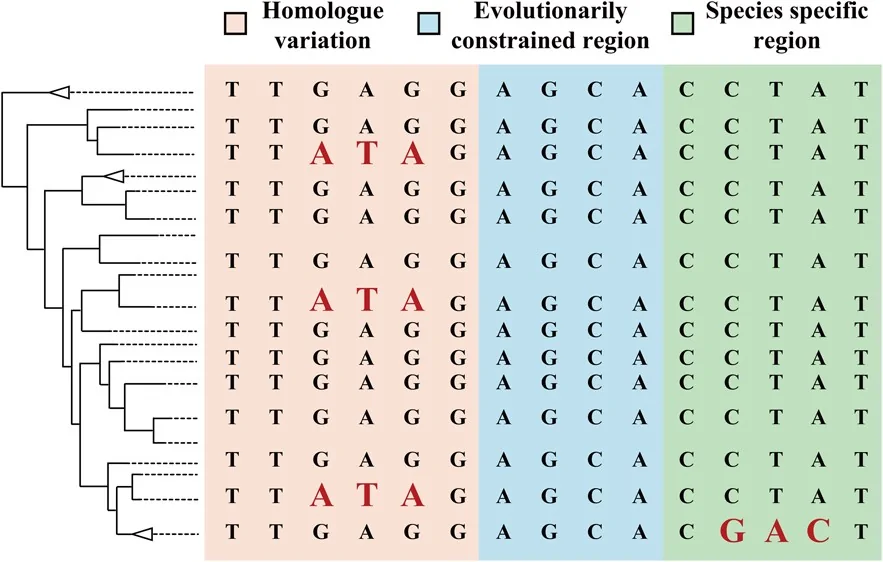
Uncovering functional tomato genes through a Solanaceae evolutionary lens. Homolog divergences (red big variants : G-A-G VS A-T-A) reveal sub/neofunctionalized genes; evolutionarily constrained regions encode core biological functions maintained across species; Species-specific regions (red big variants :C-T-A VS G-A-C) are associated with species-specific function.

Tomato and its wild relatives all belong to the Lycopersicon section of the genus *Solanum* within the Solanaceae family [13–15]. Comparative genomic analyses of these related species provide valuable insights into the evolutionary forces that have shaped the genome under natural selection [16–21], which hold potential for overcoming linkage drag. By examining sequence conservation and divergence among these taxa, homologous genes and evolutionarily conserved functional elements can be identified, thereby facilitating more accurate annotation and functional characterization of the tomato genome [16]. Moreover, such studies offer a framework for translating findings across related Solanaceous crops, enabling genome-wide identification of functional elements (Fig. 3).

**Figure 4:**
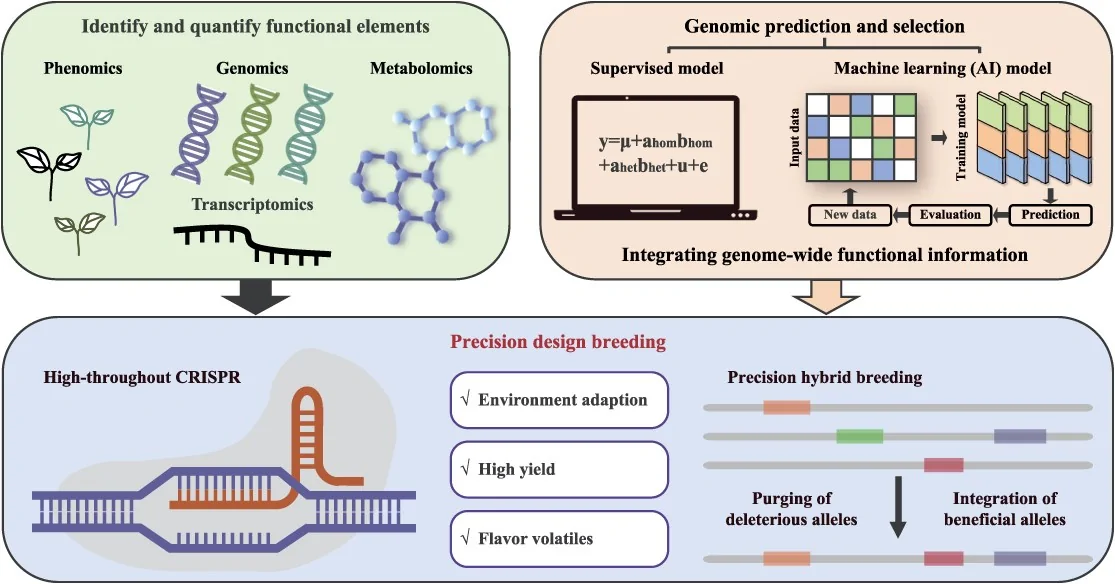
Accelerated tomato breeding through precision design breeding, enabled by genomic prediction and selection that integrate whole-genome information with supervised or AI-driven modeling. This framework enables precise identification of beneficial alleles and deleterious alleles, enhances genomic prediction and selection, and supports informed decision-making for genome editing or precision hybrid breeding, thereby accelerating trait improvement.

The goals of modern tomato breeding have shifted markedly, with increasing emphasis on consumer preferences for superior flavor, nutrition, and visual qualities. Meeting these demands requires more efficient and precise breeding strategies. Genomic design breeding offers a transformative and promising approach [22–26]. This approach relies on high-resolution genome decoding and systematic integration of functional genetic information (Fig. 4). Key strategies include: (i) leveraging comparative genomics across Solanaceous species and wild tomato relatives to uncover evolutionary insights and pinpoint functional genes [16, 18, 21]; (ii) exploiting population genetics of domestication and improvement to identify valuable alleles [1–3, 7, 27]; and (iii) applying genomic prediction and selection combined with artificial intelligence (AI) to effectively integrate both major- and minor-effect loci (Fig. 4) [28]. The systematic redomestication: by uniting comprehensive genome annotation with advanced computational modeling, precision genome design can accelerate genome-wide discovery and deployment of beneficial alleles, thereby speeding up the development of elite cultivars (Fig. 4). Ultimately, integrated genomic design breeding will be essential for enhancing yield stability, nutritional quality, and flavor in future tomato production.

## High-quality genomes facilitate tomato breeding

A comprehensive understanding of the tomato genome is fundamental to advancing both basic research and applied breeding [29–31]. Over the past decade, breakthroughs in third-generation sequencing (e.g. PacBio HiFi, Oxford Nanopore) and assembly algorithms (e.g. Hi-C scaffolding, trio-binning) have revolutionized genome assembly, leading to the production of numerous high-quality, chromosome-scale tomato genomes (Table S1) [4, 32–42]. These genomic resources provide a critical foundation for systematic investigations of gene function, trait dissection, and molecular breeding.

The release of the ‘Heinz 1706’ reference genome marked a milestone in tomato genomics, establishing the first comprehensive framework for genetic studies [30]. While this single reference laid the foundation for subsequent analyses, its limitations became increasingly evident over time. Through iterative versions (now V6), continuous improvements in gap filling and annotation refinement have greatly enhanced the completeness and accuracy of the ‘Heinz 1706’ genome, maintaining its position as a cornerstone resource for both basic research and precision breeding [34, 37, 43]. Nevertheless, reliance on a single reference genome presents inherent constraints: (i) inability to capture structural variations (SVs) and presence–absence variations (PAVs); (ii) bias against nonreference haplotypes; and (iii) limited representation of genetic diversity from wild relatives. To address these limitations, genome assembly of multiple wild tomato species have been reported, including *S. pimpinellifolium*, *S. peruvianum, S. habrochaites et al*., [4, 37, 40, 44–46]. These efforts have revealed key SVs regulating important agronomic traits such as disease resistance and fruit quality [3, 33, 42, 46], and demonstrated that wild species genomes not only compensate for the diversity gap in the reference genome but also provide valuable genetic elements for improving yield, flavor, and resistance.

Despite significant advances, dispensable genes remain underrepresented in single-reference genomes. Pangenomes constructed by integrating multiple high-quality genomes representing major subpopulations within a species or related species—substantially reduce reference bias in variant detection, uncover SVs missed by a single reference, and enable precise quantification of gene PAVs linked to important traits [47–55]. Specifically, population-scale *de novo* assemblies in tomato have facilitated the systematic discovery of SVs and PAVs, enabling robust genotype–phenotype association studies for agriculturally important traits such as disease resistance, stress adaptation, and fruit flavor [3, 4, 42]. For example, pangenome analysis revealed a ~4-kb SV (substitution) in the promoter region of *TomLoxC*, which regulates apocarotenoid biosynthesis, a key determinant of tomato flavor [3]. The alternative allele of this SV is present in 91.2% of the wild progenitor (*S. pimpinellifolium*) but only 2.2% of heirloom varieties, demonstrating how pan-genomics can help detect valuable alleles that have been lost during domestication. Similarly, long-read sequencing of a 100-genome tomato panel identified numerous SVs, demonstrating that gene dosage-altering SVs modulate expression levels to influence key agronomic traits including fruit flavor, size, and yield [42]. Although linear pangenome assemblies improve SV identification compared with single-reference genomes, they still limited the capture of complex haplotype diversity, and persistent reference bias continues to affect variant detection accuracy, particularly for population-specific alleles [56].

Graph-based approaches are now emerging to address the limitations of linear reference genomes by integrating sequences from multiple representative individuals into a unified graph framework [57, 58]. Such graph-based genome frameworks enable comprehensive SV detection, facilitating SV-based GWAS and capturing missing heritability across both wild and cultivated tomatoes [34]. This approach has revealed previously hidden genetic diversity relevant to flavor and yield improvement that was inaccessible using linear reference genomes. For example, construction of graph-based pangenome references increased explained heritability by 24% compared with linear references [34] and enabled the discovery of novel genes associated with yield and flavor-related metabolites [4]. Collectively, these studies establish graph pangenomes as essential tools for unlocking the full genetic potential of tomato.

Advances in sequencing technologies and bioinformatics have transformed crop population genomics, evolving from reliance on a single-reference genome with resequencing, to linear pangenomes, and most recently, to graph-based pangenome representations [29, 31]. This shift from linear to graph-based frameworks represents a major step toward capturing the full spectrum of genetic diversity and exemplifies how reference genomes have advanced from foundational resources to comprehensive diversity platforms, enabling both biological discovery and breeding innovation. Looking ahead, efforts should focus on producing T2T genomes and pan-transcriptome across developmental time combined with functional data, which have been reported in other species [59–63]. Together, these will lay the groundwork for precision breeding and cultivar-specific genome design.

## Multiomics insights into tomato domestication and breeding: achievements and trade-offs

### Achievements during domestication and improvement

Advances in population-scale genomic analyses have revolutionized our understanding of crop domestication histories and enabled the identification of beneficial alleles from ancestral populations [3, 5, 64]. Comparative genomic analysis of 360 tomato accessions has systematically decoded the genetic architecture underlying tomato domestication and modern breeding [5]. Artificial selection during tomato domestication and improvement has favored alleles conferring higher yield, extended shelf life, and improved disease resistance, while simultaneously depleting genetic variation in flavor-associated metabolic pathways [1, 2]. Lin *et al*. identified selective sweeps covering 8.3% (64.6 Mb) and 7.0% (54.5 Mb) of the genome during domestication and improvement, respectively [5]. These regions showed strong signals of purifying selection, providing candidate genes linked to yield, flavor, and disease-resistance traits (Fig. 2A). Notably, 18 fruit mass-related quantitative trait locus (QTLs) under selection collectively enabled the dramatic ∼100-fold increase in fruit size observed in modern tomatoes compared to their wild ancestor *S. pimpinellifolium* [5].

### Trade-offs during domestication and improvement

While artificial selection for improved yield, shelf life, and disease resistance has greatly enhanced productivity, these gains have frequently come at the expense of flavor and nutritional quality [65]. A large number of key flavor metabolites, including sugars, organic acids, amino acids, and volatiles are markedly reduced in modern commercial cultivars, with fruit weight showing a strong negative correlation with fruit quality [1]. This reflects a fundamental trade-off between yield and quality traits. The most prominent trade-off involves fruit size and sugar content [66]. For instance, large-fruited commercial varieties often exhibit reduced sugar accumulation due to low activity of sugar transporters such as *Lin5* [66]. Another striking example of this trade-off is the rare *TomLoxC* promoter allele that was selectively lost during domestication. QTL mapping and transgenic complementation have shown that *TomLoxC* modulates apocarotenoid biosynthesis, thereby enhancing the production of volatile compounds critical for tomato flavor [3]. Collectively, these findings demonstrate how modern breeding practices have profoundly reshaped the tomato metabolome, leading to a significant decline in flavor quality and nutritional value.

### Theory of the trade-offs

The yield-quality trade-off in tomato arose from a combination of domestication bottlenecks and strong directional selection for yield-related traits. This process can be attributed to the following four major mechanisms: (i) linkage drag: physical linkage of loci on chromosomes often bundles yield-enhancing alleles with quality-reducing variants (Fig. 2B) [67–69]. During domestication, genetic hitchhiking propagated the fixation of yield-associated alleles with antagonistic effects on flavor–compound synthesis; for example, selection at the *fw11.3* locus for increased fruit mass during domestication concurrently altered numerous metabolites. Specifically, eight metabolites showed divergent levels in two near-isogenic lines (NILs) differing at this locus. The fact that these same metabolites were not significantly altered in transgenic plants suggests that their changes may be driven by selection at *fw11.3* and its linked genetic background [2]. Another striking example is provided by *Lin5*: overexpression experiments have confirmed that the allele encoding Asp enhances fruit sugar accumulation, yet this allele has been almost entirely eliminated in BIG cultivar tomato [1]. Besides, *Lin5* located in a selective sweep during domestication and improvement. Together, the negative correlation between sugar content and fruit weight in *S. lycopersicum* is likely associated with the erosion of high-sugar alleles during domestication and improvement, as the preferential selection focused on larger fruits [1]. (ii) Genetic erosion via neutral processes: population bottlenecks and random genetic drift, compounded by direct selection during domestication and improvement, led to the loss of genetic diversity and, inadvertently, the loss of flavor-associated alleles (Fig. 2C) [70, 71]; and (iii) antagonistic pleiotropy: yield-enhancing mutations sometimes simultaneously suppressed the biosynthesis of flavor compounds, collectively eliminating key metabolic pathways during domestication and subsequent crop improvement (Fig. 2D). (iv) Source limitation and carbon dilution: as a source-limited plant, tomato yield is ultimately capped by total photosynthetic output. Selection for larger fruit often dilutes fixed carbon and other nutrients into increased water volume and biomass, leading to a decrease in the concentration of sugars, acids, and volatiles per unit weight, a physiological ‘dilution effect’ (Fig. 2E) [72]. The inadvertent fixation of yield-associated alleles therefore exhibits dual functions, producing pleiotropic negative effects on quality. This triad of mechanisms, hitchhiking, neutral loss, and pleiotropy, explains why modern cultivars achieve high yields at the expense of flavor complexity.

### Roads to breaking the trade-offs

Interestingly, during domestication and selection, while there existed trade-offs between yield and quality, some alleles associated with flavor traits became fixed in tomato populations due to their genetic linkage with yield-related loci [27, 73]. A striking example is *Sl-LIP8*, a key enzyme in the fatty acid-derived volatile organic compound biosynthesis pathway that generates flavor metabolites influencing consumer preference. The fixation of this allele likely occurred through genetic hitchhiking with a physically linked locus controlling fruit size—a trait under strong artificial selection during tomato domestication [73]. Another notable example involves the recently identified sugar brake genes *SlCDPK27* and *SlCDPK26* [27]. Knockout of these genes increases glucose and fructose contents by 30% while maintaining optimal fruit size and yield [27]. These cases demonstrate that flavor-related beneficial alleles can be reintroduced without decreasing fruit weight. By reintroducing heirloom or wild alleles associated with higher sugar content, balanced acidity, and enriched volatiles, it is possible to revitalize flavor in modern tomatoes while maintaining agronomic performance. This provides a roadmap for flavor restoration through marker-assisted breeding or genome editing of key QTLs controlling flavor chemistry. The key challenge is to identify functional alleles and break their linkage drags with deleterious or yield-reducing alleles.

## Evolutionary lens-powered tomato genetics and breeding

High-efficiency and high-accuracy identification of beneficial and deleterious alleles is crucial for precision breeding in tomato. However, the resolution of functional loci during domestication has been limited by population bottlenecks, population structure, and linkage drag [74]. To overcome these limitations, insights from evolutionary processes operating over longer timescales [75–87], characterized by accumulated genetic substitutions and extended periods of selection, can help overcome the constraints imposed by linkage drag and population structure (Fig. 3).

The Solanaceae family, which includes economically important crops such as eggplant (*S. melongena*), potato (*S. tuberosum*), and pepper (*Capsicum annuum*), in addition to tomato, is one of the most species-rich and ecologically diverse plant families. The backbone phylogeny of Solanum and Solanaceae provides strong support for its major clades [18, 19, 21]. Furthermore, the current availability of >100 Solanaceae genomes has revolutionized functional gene discovery, elucidations of trait evolution mechanisms, and breeding applications [21]. Recent advances in genome sequencing and bioinformatics tools for comparative genomics now enable comprehensive identification of: (i) genome-wide paralogous and orthologous gene pairs, (ii) evolutionarily conserved coding and noncoding regions, and (iii) rapidly evolving or species-specific elements, which are often associated with lineage-specific phenotypic traits [17, 20, 88–92]. These developments provide unprecedented opportunities to trace the evolution of functional elements and decipher their biological significance. As a model vegetable crop within this family, tomato will benefit tremendously from comparative genomic approaches.

Homologous gene diversification over short evolutionary timescales represents a critical yet underexplored dimension of trait evolvability [16]. By characterizing homolog diversification patterns at the genus or family level, functionally redundant gene copies that may serve as genetic buffers stabilizing key agronomic traits during breeding can be identified [16]. Systematic identification and integration of these homolog variations are therefore prerequisites for robust cross-species genotype–phenotype extrapolation. A deeper understanding of such rapid homolog evolution is essential for developing robust cross-species frameworks to predict phenotypic outcomes from genetic variation. For example, analysis of a pangenome constructed from genomes of 22 distinct *Solanum* species revealed widespread variation in a set of 150 genes and their associated paralogue affecting 16 domestication and breeding traits, including 17 orthogroups related to three major components of crop domestication syndromes: (i) flowering time and plant architecture, (ii) inflorescence architecture, and (iii) flower number and fruit size [16]. A notable case involves variation of *CLAVATA3* (*CLV3*), a gene associated with fruit locule number. In tomato, a promoter SV in this gene reduced *CLV3* expression, leading to the formation of additional locules. Evolutionary events involving *CLV3* homologs, such as the loss of an ancestral pseudogenized copy associated with locule number variation in eggplant, further illustrate this dynamic. *CLE9*, a partially redundant homolog of *CLV3*, partially suppresses the increased locule number. Across the Solanum pangenome, *CLE9* is either absent or pseudogenized, except in tomato and *S. americanum*. Taken together, homolog variation—including pseudogenization and independent tandem duplications of *CLV3*, as well as the emergence and subsequent loss of *CLE9*—underlines the natural variation in fruit locule number. Collectively, these findings demonstrate that comparative analysis of homolog variation across genera or families not only facilitates the discovery of favorable alleles but also provides a powerful framework for crop improvement.

Evolutionarily conserved sites are genomic regions that retain high sequence conservation across distantly related species due to strong selective pressure acting over long evolutionary timescales [79, 80, 82]. These regions typically correspond to functionally important elements and exhibit conserved biological activity (transferability) across related species. Comprehensive phylogenies with sufficient neutral site substitutions, derived from genome-wide sequence alignments, are essential for reliably predicting evolutionary constraints, often quantified as the number of rejected substitutions. In Solanaceae, phylogenomic analyses have identified evolutionarily constrained coding and noncoding elements, facilitating the discovery of functional elements and genes that have directly contributed to advances in potato breeding [21]. This approach also holds great promise for improving tomato breeding. Through systematic identification and characterization of phylogenetically conserved sequences and lineage-specific deletions or insertions in tomato, functionally significant genomic elements can be discovered. Such findings establish a novel framework for elucidating how sequence variation drives biological specialization in tomato and other Solanaceous crops.

## Perspective: increasing genetic gains and advancing precision breeding

The divergence between historical selection targets (fitness, yield, storage) and contemporary consumer demands (flavor, nutrition) necessitates a paradigm shift in tomato breeding—essentially a process of ‘redomestication’ under modern agronomic and climatic conditions [93]. Achieving precision design breeding to increase genetic gain requires systematic identification, validation, and integration of genome-wide functional genetic elements. With genome resequencing data now readily available, combining high-throughput phenotyping with advanced analytical frameworks, such as evolutionary lens analysis, artificial selection studies and machine learning, enables accurate prediction and functional annotation of individual genomic sites. These foundations pave the way for constructing ideal genomes and even personalized genomes through precision genome design, guided by genomic prediction using supervised or AI modeling (Fig. 4). Looking ahead, massively parallel, high-accuracy editing of hundreds of loci per generation as well as precision hybrid breeding will accelerate development of tomato cultivars with enhanced agronomic performance and consumer-oriented traits. In the coming decades, efforts will increasingly focus on the integration of genome-wide functional information into supervised or AI-driven models, advancing genomic prediction and selection, and ultimately accelerating precision design breeding (Fig. 4).

### Comprehensive genome-wide functional annotation and genetic dissection

A prerequisite for precision breeding is the comprehensive annotation of functional sites across the entire genome, enabling optimal configurations of known alleles into favorable combinations. Dissecting regions or genes shaped by natural or artificial selection during evolution, domestication, and improvement enables efficient and precise identifications of functional genes and alleles. Recent technological advances, including single-cell omics [94], CRISPR-based (clustered regularly interspaced short palindromic repeats) functional screens [95], and deep learning models such as AlphaFold or gene prediction tools [96], enable systematic annotation of all functional elements in the genome (e.g. coding/noncoding genes, enhancers, promoters, and regulatory motifs). For instance, the recently developed DNA sequence model, AlphaGenome, has established a unified framework capable of directly predicting the activities of diverse genomic functional elements from DNA sequences, while simultaneously predicting key modalities such as gene expression, chromatin accessibility, 3D conformation, and RNA splicing [96]. Trained on human and mouse genomes, AlphaGenome matched or exceeded the performance of current best-in-class external models in 25 out of 26 variant effect prediction benchmarks, representing a significant advance in deep learning for ‘sequence-to-function’ prediction [96]. Epigenomic profiling approaches (e.g. ChIP-seq, ATAC-seq, DNA methylation analysis) further delineate active regulatory regions [97–99]. By integrating these resources with GWAS, functional genetic variants can be rationally combined with unprecedented speed and accuracy [100]. Moreover, saturation mutagenesis approaches such as deep mutational scanning can systematically assess the phenotypic impact of nearly all possible genetic variants [101]. At the same time, CRISPR-based perturbation screens (CRISPRi/a, base editing, prime editing) provide scalable functional validation of candidate loci [102]. Together, these advances provide the foundation for genome-powered tomato breeding, where functional elements can be precisely designed and assembled to optimize yield, resilience, flavor, and nutrition.

Modern breeding, however, requires a bidirectional strategy: systematically identifying and pyramiding beneficial alleles while simultaneously purging deleterious genetic variants, a strategy essential for sustainable crop improvement. Although substantial progress has been made in elite allele discovery [3, 27, 41, 42, 66, 103–123], systematic characterization of deleterious alleles has received far less attention in tomato. The impact of such deleterious alleles is not unique to tomato. For instance, in potato, the deleterious alleles severely decrease fitness, leading to the reduction in yield and directly impeding the development of vigorous inbred lines [21, 124, 125]. Similarly, in maize, numerous small-effect deleterious variants persist within elite breeding pools, although individually subtle, they collectively contribute to background mutational load, which can compromise traits like general vigor, leading to a negative association with yield-related traits [126–130]. Methods based on evolutionary conservation and protein function (e.g. GERP++, SIFT, PolyPhen) predict the likelihood of variants being deleterious [131–133]. Furthermore, integrative machine learning approaches (e.g. CADD, Eigen) are increasingly used to prioritize deleterious variants by synthesizing diverse genomic and functional data layers [134, 135]. These tools are particularly adept at detecting not only large-effect mutations but also variants with subtle effects. In tomato inbreds, the lethal deleterious variants have been purged, while relatively minor deleterious variants accumulate and collectively decrease fitness [136]. Approaches such as GWAS have primarily targeted major-effect loci [1, 4, 137, 138], largely overlooking the cumulative impact of small-effect deleterious variants. However, these variants collectively contribute to mutational load, which can decrease fitness-related traits such as yield [21, 126]. Hence, combining these prediction tools with genome-wide functional annotation of both beneficial and deleterious alleles can help unlock hidden genetic variance for breeding, reduce mutational load in elite varieties, and ultimately design genomes that balance productivity, resilience, and quality. This shift in focus, treating both beneficial and deleterious alleles as central to breeding targets, will be critical for accelerating genetic gain in tomato.

### Recapturing genetic gain through genomic prediction and selection

Genomic prediction (GP) and selection (GS) have proven highly effective in enhancing genetic gain, fundamentally transforming contemporary breeding programs across animals and plants species [139–141]. GS models integrate both major-effect loci and genome-wide markers with minor effects, employing supervised or machine learning (AI-driven) approaches to predict breeding values and guide selection decisions [28, 142]. In animal breeding, genomic selection has fundamentally changed industry standards. By replacing traditional progeny testing, it has slashed the dairy cattle breeding generation interval from ~5 to ~2 years, dramatically accelerating genetic gain [139]. For instance, in the Netherlands, the adoption of genomic selection in 2008 increased the annual rate of genetic progress for on-farm production by 60% (from 21 to 34 index points per year) [139]. In crops such as maize and rice, genomic prediction is now a routine tool to accelerate breeding cycles in major international seed companies. By applying GS models, breeders can predict adult plant phenotypes to guide selection of superior lines years earlier than waiting for results from conventional multilocation field trials [28, 142]. This framework supports the design of optimal allele combinations for traits such as yield across diverse environments.

AI/ML approaches are now widely deployed for high-throughput phenotyping, and the development of predictive models for complex traits [143–145]. AI-driven image analysis and sensor-based phenotyping can be applied in field or greenhouse trials to automatically and objectively measure traits such as fruit morphology, vine architecture, and biotic/abiotic stress responses, generating the high-quality data needed to power GP models [143]. For example, AI/ML techniques have demonstrated broad applicability in plant phenotyping across a range of crops, including but not limited to soybean, maize, wheat, and rice [146–149]. Specific applications encompass leaf counting, estimation of leaf area index and aboveground biomass, root segmentation, spike number quantification, measurement of nitrogen and chlorophyll content, as well as the classification and quantification of biotic and abiotic stresses [150–164]. By constructing a training population with genotypic and phenotypic data, AI/ML can be used to build robust prediction models for traits such as yield, fruit quality, and stress tolerance, enabling rapid selection in early breeding generations [143, 165]. Through the integration of multiomics data types, such as gene ontology, transcriptomic, and GWAS data, AI has been validated to enhance genomic prediction accuracy in key plant species including *Arabidopsis thaliana*, maize, rice, soybean, and wheat [143, 166–169]. The integration of genomic prediction with AI/ML-powered phenotyping and model development accelerating the development of genomic prediction and selection.

The core methodology is fully transferable. Successful application in tomato primarily hinges on building a robust, species-specific training population with high-density genotypic and high-fidelity phenotypic data, as the predictive algorithms themselves are universal. The main challenges are not conceptual but practical, relating to data generation, integration of automated phenotyping, and resource allocation [170]. By adopting and adapting this proven paradigm, tomato breeding can achieve similarly accelerated rates of genetic gain for yield and fruit quality.

### High-throughput CRISPR

The advent of CRISPR-Cas9 genome editing has ushered in a transformative era for plant biology, enabling unprecedented precision in genetic manipulation [171–173]. CRISPR-Cas9 genome editing is revolutionizing tomato breeding by enabling precise optimization of complex trait architectures. In principle, this technology could facilitate simultaneous enhancement of multiple trait categories: (i) yield potential through modulating regulators of meristem size and determinants of fruit locule number [104, 174, 175]. Previous research has demonstrated that epistatic interactions within the *CLV-WUS* pathway facilitate synergistic enhancement of fruit locule number by the fas and lc loci [175]. Using CRISPR-Cas9-mediated targeting of the *CArG cis*-regulatory element in the *SlWUS* gene enabled the generation of an engineered allele that functionally recapitulates the natural *lc* variant. In *S. pimpinellifolium*, edited lines exhibited a multilocular fruit frequency of 76%, significantly exceeding the 43% observed in fas monogenic controls and phenotypically aligning with near-isogenic lines harboring the native *lc* allele [175]. (ii) fruit quality via targeted modification of candidate genes controlling flavor and texture [103, 105, 176, 177]; the prior investigation confirmed the function of *FIS1* in near-isogenic lines, demonstrating significantly enhanced compression resistance in its knockout mutants [103]. Furthermore, CRISPR-mediated *FIS1* knockout mutants were generated in two distinct genetic backgrounds, wild currant tomato and cultivated cherry tomato, all of which consistently exhibited superior agronomic traits, including increased fruit firmness, thickened cuticle, and extended shelf life, without compromising other critical fruit characteristics such as fruit weight, flavor, color, or ripening time [103]; and (iii) abiotic and biotic stress tolerance by editing osmotic regulators and other stress-responsive genes [178–182]. For example, CRISPR-Cas9-mediated knockout of *SlBBX18* significantly enhanced both physiological adaptation and agronomic stability in tomato under drought stress [178]. Importantly, CRISPR offers a potential route for direct improvement of flavor volatiles and nutritional content without linkage drag, thereby aiming to overcome trade-offs between productivity and quality.

However, realizing this potential faces significant challenges. These include inefficient gene upmodulation, potential off-target effects, and delivery systems with variable efficiency across species [183]. Moreover, our capacity to design effective multiplexed edits for dozens of targets is currently limited by biological understanding far more than by technical feasibility. Looking forward, with the rapid advances of genome editing technologies, massively parallel, high-precision modifications across hundreds of genomic loci may become attainable. In the future, multiplexed editing systems could aim to achieve modifications of numerous loci per generation in tomato, which, if realized, could accelerate the development of consumer-preferred varieties that would require decades of traditional breeding.

### Integrated genomic design

The convergence of genomic prediction, genome design, and precision breeding is poised to transform tomato improvement into a predictive science. By integrating (i) genome-wide functional annotation, including evolutionarily constrained sites, selective sweeps, and *cis*-regulatory elements; (ii) genomic prediction and selection through supervised or AI-driven trait modeling, enabling the prediction of yield, resilience, and flavor-related metabolites across diverse environments with tomato diversity panels; (iii) CRISPR-Cas mediated multiplex editing, enabling the simultaneous modification of >100 loci per breeding cycle; and (iv) precision hybrid breeding to optimize parental selection and targeted crossing for beneficial allele aggregation, which allows breeders to engineer ‘designer genomes’ with unprecedented efficiency and specificity [184], thereby accelerating the development of superior tomato varieties (Fig. 4). This paradigm shift enables the development of environment-adapted cultivars with stable, high yields across diverse agroecosystems and consumer-tailored varieties with precisely tuned flavor and nutrition profiles, free from linkage drag. This has been partially applied to other crops. For example, guided by structural information of *GmSWEET10a/b* through AlphaFold [185], targeted CRISPR-Cas9 editing was employed to create bespoke allelic variants not observed in the natural germplasm pool. Subsequent multiyear, multilocation field trials confirmed that the engineered lines exhibited stable, statistically significant increases in seed oil content, crucially without incurring any yield penalty [185]. This work exemplifies a shift from phenotype-based selection to an AI-driven, structure-informed precision design paradigm in crop breeding. However, there are still significant technical and practical bottlenecks for application in tomato breeding. The genome-wide functional annotation is currently constrained by large gaps in functional annotation, especially for noncoding regulatory regions. Genomic prediction accuracy and scalability are often limited by the need for massive, high-quality phenotyping data across diverse environments. Nevertheless, incremental advances are being made in tomato genetic and breeding. For example, in tomato, the development of genomic prediction models [186–189] and AI-assisted robotic systems aims to automate cross-pollination and streamline hybrid breeding workflows [190]. These technological developments offer practical pathways toward sustainable precision agriculture. Ultimately, integrated genomic design offers a sustainable framework for feeding a projected 10 billion people by 2100 under the pressures of climate change [191].

#### Dedication

This review is dedicated to Prof. J.J. Giovannoni, a pioneering scientist whose groundbreaking work on the molecular and genetic regulation of tomato fruit ripening has profoundly advanced our understanding of plant development and signal transduction. His innovative research—including the positional cloning of key ripening genes, elucidation of ethylene- and light-responsive pathways, and major contributions to tomato genomics—has provided foundational insights that continue to shape the field today. Prof. Giovannoni’s rigorous scholarship, mentorship, and collaborative spirit continue to inspire and guide generations of researchers. We extend our deepest gratitude for his transformative contributions to plant biology and his enduring leadership in advancing the science of crop improvement.

## Supplementary Material

Web_Material_uhag172
